# Supplementary material for: Predictors of Growth of Vestibular Schwannoma After Gamma Knife Treatment: A Systematic Review
Source: Cancers (Basel). 2025 Jun 14;17(12):1993. doi: 10.3390/cancers17121993 (PMC12191170; doi:10.3390/cancers17121993)
Supplement: Supplementary file 1 [file cancers-17-01993-s001.zip › Table S1.pdf]

Table S1: This table presents the NOS scale scores of 9 studies, which assess the risk of bias by assessing the quality of cohort studies in three aspects: selectivity, comparability, and outcome.

Reference	Selection				Comparability	Outcome			Score
	Representativeness of the Exposed Cohort	Selection of the Nonexposed Cohort	Ascertainment of intervention	Demonstration that Outcome of Interest Was Not Present at Start of Study	Comparability of Cohorts on the Basis of the Design or Analysis	Assessment of Outcomes	Was Follow-Up Long Enough for Outcomes to Occur	Adequacy of Follow Up to Cohorts	
Grzegorz Turek [9], 2023	★	★	★	☆	★☆	★	★	★	7
Ferdinand C. A. Timmer [10], 2011	★	★	★	☆	★★	★	★	★	8
Alexander P. Marston [11], 2017	★	★	★	☆	★★	★	★	☆	7
Theresa Wangerid [12], 2014	★	★	★	☆	★☆	★	★	★	7
Stephen Johnson [13], 2019	★	★	★	☆	★★	★	★	★	8
Rick van de Langenberg [14], 2011	★	★	★	☆	★★	★	★	☆	7
Chih-Chun Wu [15], 2017	★	★	★	☆	★★	★	☆	☆	6
Soroush Larjani [16], 2014	★	★	★	☆	★★	★	★	★	8
Stijn Klijn [17]	★	★	★	☆	★★	★	★	★	8

The **black stars** represent the scores obtained, while the **white stars** represent the scores not obtained. The total score is 9 points, with a score greater than 7 indicating high-quality research.
