# Supplementary material for: Predictors of Growth of Vestibular Schwannoma After Gamma Knife Treatment: A Systematic Review
Source: Cancers (Basel). 2025 Jun 14;17(12):1993. doi: 10.3390/cancers17121993 (PMC12191170; doi:10.3390/cancers17121993)
Supplement: Supplementary file 1 [file cancers-17-01993-s001.zip › Table S2.pdf]

Table S2: Radiation dose of nine studies.

Study Number	Author, Year	Margin	Max	coclear	mean
1	Grzegorz Turek [9], 2023	12(11.5-13)	/	2.3（0.7-7.8）	/
2	Ferdinand C. A. Timmer [10], 2011	11(9.3-12.5)	19.9 (16.0–25.5)	/	/
3	Alexander P. Marston [11], 2017	13（12-14）	26（24-28）	/	/
4	Theresa Wangerid [12], 2014	12.3（11-16）	26(17-47)	/	/
5	Stephen Johnson [13], 2019	13（8-20）	/	/	/
6	Rick van de Langenberg [14], 2011	11.6（10.3-13）	/	/	/
7	Chih-Chun Wu [15], 2017	12（11-13）	21（15-23）	/	16.3（13.6-21.8）
8	Soroush Larjani [16], 2014	/	24.00 (22.22–30.10)	/	17.12 (15.61–19.37)
9	Stijn Klijn [17], 2016	11(11-13)	/	/	/
